# Climate, Catering, and Health

**Published:** 1903-04-25

**Authors:** 


### The Hospital.

A Journal of

The Medical Sciences and Hospital Administration.

Edited by Sir Henry Burdett, K.C.B.

Vol. XXXIV.—No. 865. [April 25, 1903.

### CLIMATE, CATERING, AND HEALTH.

Conditions of climate have more to do with health and even with life than most people realise. Persons of both sexes after 50 years of age, providing they are able to secure for themselves a maximum of sunshine and open-air life throughout the course of each year, may retain their energies and vitality unimpaired until quite an old age. Of course, diet and exercise combined with common sense are also material factors in the maintenance of energy and vigour after middle life, but in practice the more vigorous the man the more moderate is usually his appetite. Bodily health means mental vigour and energy at their best. These considerations are becoming more and more understood by men who have made their fortunes, and to them in great measure, no doubt, is due the development of many resorts in Europe where sunshine and fine weather are to be found during several months of each year. One patient, who is well over 70, explained to us the other day that he attributes his robust health and vigour to the fact that for 20 years past he has made it his business to spend 10 months of each year at least in places where sunshine and fine weather may be met with with reasonable certainty. Of course no one place will continuously yield such perfection of climate, but a little management enables the thing to be done with reasonable certainty by the more intelligent of our race.

The advantages of climate and health to which we refer are well within the means of most middle-class people. Each resort where sunshine reigns has its season, during the greater part of which accommodation may be had at a cost little, if any, greater than that entailed by continuous residence in England. We make this statement after full investigation, though it is calculated to be disbelieved at the outset by all but a few practical people who have studied the questions involved. The truth is Society—*i.e.* the great rush of pleasure seekers—all comes together to the places where sunshine is constant. So the modern tendency is to confine the period of their visitation to a few weeks instead of extending it over months as was formerly the practice. Hence hotel keepers and caterers for the amusement of the visitors, have at present to make their profits during some six weeks of each year, when the prices are necessarily prohibitive to persons of moderate means. But sunshine and fine weather prevail, not merely during the six weeks which Society selects for its incursion, but are constant, or almost constant, during five or six months of each year. It follows that prices are low, and relatively poor people are welcome as visitors during quite three fourths of the whole period of sunshine in each such resort. That is why we repeat it is a lack of intelligence rather than of cash which prevents the majority of middle-class people in England from securing for themselves the conditions of climate by a change of residence, which after middle age will best promote their physical and mental well-being and vitality.

We are writing this on a lovely day in brilliant sunshine on the Riviera. It is the 20th of April, and the ancient traditions still prevail here whereby the season is supposed to end immediately after Easter. Yet from the beginning of April to the middle of May the Riviera is at its best, a fact well known and appreciated by visitors, who are, however, forced to leave the country because of the purblind stupidity of the municipalities, the railways, and the hotel proprietors, who combine by the withdrawal of the usual facilities for amusement, travel, and accommodation, to force their guests to take their departure.

In the South of France, as elsewhere, conditions of climate have altered materially during the last ten years, but the conservatism of custom causes the authorities we have mentioned to shut their eyes to plain facts and continue to grumble at bad seasons, although their diminished profits are due mainly to their own stupidity. Instead of preparing for guests at the commencement of each November, December 1st should be chosen as the first day of each season, for a study of the climatic conditions of the South of France and England would prove that in the former case the best weather for visitors is to be met with nowadays between December 15th and May 15th in each year. In England formerly the worst months in the year were probably November, December and part of January. Of late years the worst weather has usually prevailed in February, March and April. Thus, whilst we have had this season on the Riviera during the whole of April continuous sunshine and lovely weather, in England there has been ice on the ponds, a succession of snowstorms, and weather which is generally described in the newspapers " as the most winterly experienced for some time."

It is our aim to advocate principles which tend to promote sound health and happiness for the people. In view of the facts we have just set forth it must be patent to everybody that the more intelligent of our race who have been shivering at home or in Paris this Eastertide in discontent and comparative misery, may take comfort as to the future if they decide to spend next April and part of May in the South of France. That they will do so in large numbers we have little doubt, providing the French municipalities, railways, and hotel-keepers announce to the world in the autumn that they have determined to recognise plain facts, and to provide in future for the comfort and enjoyment of their visitors from the middle of December to the middle of May each year. They should add that this decision is due to their knowledge that during the whole of that period the maximum of sunshine and the best of weather is to be found on the Riviera.

